# Dual-strain genital herpes simplex virus type 2 (HSV-2) infection in the US, Peru, and 8 countries in sub-Saharan Africa: A nested cross-sectional viral genotyping study

**DOI:** 10.1371/journal.pmed.1002475

**Published:** 2017-12-27

**Authors:** Christine Johnston, Amalia Magaret, Pavitra Roychoudhury, Alexander L. Greninger, Daniel Reeves, Joshua Schiffer, Keith R. Jerome, Cassandra Sather, Kurt Diem, Jairam R. Lingappa, Connie Celum, David M. Koelle, Anna Wald

**Affiliations:** 1 Department of Medicine, University of Washington, Seattle, Washington, United States of America; 2 Vaccine and Infectious Disease Division, Fred Hutchinson Cancer Research Center, Seattle, Washington, United States of America; 3 Department of Laboratory Medicine, University of Washington, Seattle, Washington, United States of America; 4 Department of Biostatistics, University of Washington, Seattle, Washington, United States of America; 5 Genomics and Bioinformatics Resource, Fred Hutchinson Cancer Research Center, Seattle, Washington, United States of America; 6 Department of Global Health, University of Washington, Seattle, Washington, United States of America; 7 Department of Pediatrics, University of Washington, Seattle, Washington, United States of America; 8 Department of Epidemiology, University of Washington, Seattle, Washington, United States of America; 9 Benaroya Research Institute, Seattle, Washington, United States of America; University of Bern, SWITZERLAND

## Abstract

**Background:**

Quantitative estimation of the extent to which the immune system’s protective effect against one herpes simplex virus type 2 (HSV-2) infection protects against infection with additional HSV-2 strains is important for understanding the potential for HSV-2 vaccine development. Using viral genotyping, we estimated the prevalence of HSV-2 dual-strain infection and identified risk factors.

**Methods and findings:**

People with and without HIV infection participating in HSV-2 natural history studies (University of Washington Virology Research Clinic) and HIV prevention trials (HIV Prevention Trials Network 039 and Partners in Prevention HSV/HIV Transmission Study) in the US, Africa, and Peru with 2 genital specimens each containing ≥10^5^ copies herpes simplex virus DNA/ml collected a median of 5 months apart (IQR: 2–11 months) were included. It is unlikely that 2 strains would be detected in the same sample simultaneously; therefore, 2 samples were required to detect dual-strain infection. We identified 85 HSV-2 SNPs that, in aggregate, could determine whether paired HSV-2 strains were the same or different with >90% probability. These SNPs were then used to create a customized high-throughput array-based genotyping assay. Participants were considered to be infected with more than 1 strain of HSV-2 if their samples differed by ≥5 SNPs between the paired samples, and dual-strain infection was confirmed using high-throughput sequencing (HTS). We genotyped pairs of genital specimens from 459 people; 213 (46%) were men, the median age was 34 years (IQR: 27–44), and 130 (28%) were HIV seropositive. Overall, 272 (59%) people were from the US, 59 (13%) were from Peru, and 128 (28%) were from 8 countries in Africa. Of the 459 people, 18 (3.9%) met the criteria for dual-strain infection. HTS and phylogenetic analysis of paired specimens confirmed shedding of 2 distinct HSV-2 strains collected at different times in 17 pairs, giving an estimated dual-strain infection prevalence of 3.7% (95% CI = 2.0%–5.4%). Paired samples with dual-strain infection differed by a median of 274 SNPs in the U_L__U_S_ region (range 129–413). Matching our observed dual-strain infection frequency to simulated data of varying prevalences and allowing only 2 samples per person, we inferred the true prevalence of dual-strain infection to be 7%. In multivariable analysis, controlling for HIV status and continent of origin, people from Africa had a higher risk for dual-strain infection (risk ratio [RR] = 9.20, 95% CI = 2.05–41.32), as did people who were HIV seropositive (RR = 4.06, 95% CI = 1.42–11.56).

**Conclusions:**

HSV-2 dual-strain infection was detected in 3.7% of paired samples from individual participants, and was more frequent among people with HIV infection. Simulations suggest that the true prevalence of dual-strain infection is 7%. Our data indicate that naturally occurring immunity to HSV-2 may be protective against infection with a second strain. This study is limited by the inability to determine the timing of acquisition of the second strain.

## Abstract 2

In a nested cross-sectional study, Christine Johnston and colleagues examine the prevalence of dual-strain HSV-2 infection as a measure of immune system efficacy and potential for vaccine development.

## Author summary

**Why was this study done?:**

- There is a global need for an effective vaccine that prevents herpes simplex virus type 2 (HSV-2) infection.
- The authors asked: does naturally occurring immunity to HSV-2 infection prevent against infection with another HSV-2 strain (“dual-strain infection”)?
- Understanding how frequently HSV-2 dual-strain infection occurs and the risk factors for HSV-2 dual-strain infection is important for HSV-2 vaccine design.

**What did the researchers do and find?:**

- Two genital swabs containing HSV-2 were collected at 2 different time points from 459 participants enrolled in natural history studies and HIV prevention studies in the US, Peru, and Africa. These paired samples underwent a genotyping assay to determine if different HSV-2 strains were present in single individuals.
- For paired samples that appeared different at the 2 time points, high-throughput sequencing of the HSV-2 genome was performed to confirm the presence of HSV-2 dual-strain infection.
- HSV-2 dual-strain infection was found in 17 (3.7%) of 459 people. People with HIV infection had a 4-fold increased risk of dual-strain infection, and people from Africa had a 9-fold increased risk of dual-strain infection.

**What do these findings mean?:**

- In this study, HSV-2 dual-strain infection occurred infrequently, suggesting that naturally occurring HSV-2 immunity may be effective at preventing infection with a second strain.
- This study is limited by the inability to determine the timing of acquisition of the second strain and the inability to determine the clinical consequences of HSV-2 dual-strain infection.
- The genotyping assay could only be performed on samples with moderate levels of HSV-2 present, which may have led to underestimation of the frequency of dual-strain infection.

## Introduction

Development of an effective herpes simplex virus type 2 (HSV-2) prophylactic vaccine is a global priority to improve sexual health [1,2]. Although several HSV-2 subunit vaccine candidates appeared effective in animal models, these vaccines have failed to prevent HSV-2 disease in humans in Phase III clinical trials, despite eliciting high levels of neutralizing antibody [3,4]. Knowledge of HSV-2 immunology has advanced with identification of B cell and T cell epitopes as well as identification of tissue-resident memory T cells; however, correlates of HSV-2 immunity in immunocompetent people have not been identified [5–9].

A key component to understanding an “effective” immune response is knowledge of whether naturally occurring HSV-2 infection is sufficient to prevent HSV-2 infection with a heterologous strain. Recently, vaccine candidates and other herpes simplex virus (HSV) products essentially presenting the entire HSV proteome to the immune system have entered clinical trials or have been approved by the US Food and Drug Administration, such that elicitation of coordinated T and B cell responses to diverse HSV targets is now potentially within reach. For example, the replication-incompetent HSV-2 vaccine candidate dl5-29 has completed a Phase I trial [10]. The deletion of CD8 T cell immune evasion genes from the replication-competent HSV-1 oncolytic virus that is approved for melanoma therapy raises the possibility that whole virus vaccines might safely elicit responses that are stronger than those afforded by natural infection [11]. Now that elicitation of diverse immune responses to HSV-2 is becoming possible, it is increasingly important to quantify the extent of protection afforded by HSV-2 infection against infection with a second strain.

In this study, we define shedding of 2 different HSV-2 strains at 2 different time points as “dual-strain infection.” This definition does not address whether strains were acquired simultaneously at the time of primary infection or sequentially (defined as “superinfection” in the HIV field) [12]. HSV-2 dual-strain infection has been explored in prior studies with genotyping based on restriction length polymorphism (RFLP) analysis [13–15]. Later studies used PCR-based polymorphisms in regions with variable numbers of repeats; these studies were performed in a small number of people but showed striking differences in dual-strain infection in those who were HIV seronegative (1 of 8, 12.5%) and HIV seropositive (11 of 11, 100%) [16]. There are no standardized methods for performing HSV-2 genotyping, and studies exploring dual-strain infection have seldom been performed using more modern techniques that are able to query a substantial proportion of the genome. To our knowledge, previous studies of HSV-2 dual-strain infection have also investigated relatively small numbers of participants.

We recently sequenced 38 HSV-2 genomes from genital swabs obtained from individuals in the US, Peru, and several countries in Africa [17]. From these sequences, we identified single nucleotide polymorphisms (SNPs) that could best differentiate between samples to design a novel custom genotyping panel using an array-based genotyping assay. Such assays have previously been customized to type *Salmonella typhi* and *Vibrio vulnificus*, as well as *Plasmodium falciparum* [18–20]. To our knowledge, this is the first application of an array-based assay for HSV genotyping. We developed the custom genotyping platform to determine the prevalence of HSV-2 dual-strain infection and to identify risk factors for dual-strain infection. Women have a 2-fold increased risk of HSV-2 seropositivity compared to men, and people with HIV infection also have a higher rate of HSV-2 seropositivity than the general population [21,22]. We hypothesized that women, people with HIV infection, and people from regions with high seroprevalence of HSV-2 infection would have increased risk of HSV-2 dual-strain infection.

## Methods

### Ethics

Written informed consent was obtained from all participants, and procedures were approved by the University of Washington Human Subjects Division.

### Study design

We conducted a cross-sectional study, nested within genital HSV-2 natural history studies conducted at the University of Washington Virology Research Clinic (UW-VRC) between 1 January 1993 and 31 December 2014 and 2 HIV prevention clinical trials conducted in Peru and several African countries (HIV Prevention Trials Network [HPTN] 039, ClinicalTrials.gov NCT00076232, and the Partners in Prevention HSV/HIV Transmission Study, ClinicalTrials.gov NCT00194519) [23,24]. The eligibility criterion for this study was the availability of at least 2 genital swab specimens containing ≥10^5^ copies HSV DNA/ml taken at least 1 day apart (S1 Fig). At minimum, 2 samples are required to estimate the prevalence of dual-strain infection because it is unlikely that 2 distinct strains would be shed at the same time and at the same location in high enough quantities to be differentiated from each other.

### Samples

Of those eligible, people were selected to attempt to equally balance sex, to have 25% of the sample with HIV infection, and to have 25% from Africa and 25% from Peru and 50% from the US. Samples were collected a median of 5 months apart (IQR: 2–11 months); whenever possible, samples from the UW-VRC were prioritized based on longest length of time between sample collections. Baseline demographic and health information including sex, age, HIV status, number of sexual partners since initiation of sexual activity, type of sexual partnership (heterosexual or men who have sex with men), and continent of origin was collected at enrollment and included in the univariable and multivariable analysis.

### Selection of SNPs for HSV-2 genotyping assay

SNPs in the HSV-2 genome were identified by performing high-throughput sequencing (HTS) of genital HSV-2 swabs from 38 people as previously described [17]. Population-prevalent SNPs, defined as SNPs present in at least 10% and at most 90% of specimens, were evaluated for inclusion into the genotyping assay. To determine the optimal SNPs to differentiate between HSV-2 strains, a cluster analysis was performed by computing distance as an absolute difference between the sequences (Manhattan method) using “hclust” in R [25] (S1 Fig). The “FasTagger” function (version 3.0), which reduces the number of SNPs to distinguish sequences through evaluation of multilocus disequilibrium, was used to rank the SNPs according to their ability to distinguish sequences [26]. This analysis identified 96 SNPs to diagnose HSV-2 strains as the same or different (S1 Table).

### Genotyping assay

A custom array-based genotyping assay (GoldenGate, Illumina) was designed using the SNPs identified in S1 Table. DNA was extracted from genital samples using QIAamp DNA Blood Kit (Qiagen), with the following modifications: wash AW1 was omitted, and 2 washes of AW2 were performed. HSV PCR was performed as previously described [27], and samples underwent the genotyping assay as per manufacturer instructions. Briefly, samples were biotinylated and immobilized onto streptavidin-conjugated paramagnetic particles. Using the custom multiplex Oligo Pool Assay (Illumina), oligonucleotides were hybridized to DNA, and allele-specific extension and ligation was performed. The extended and ligated products formed a template that was transferred to a PCR mixture and amplified using universal primers. Next, the strand containing fluorescent signal was isolated and hybridized to Universal BeadChips and subsequently imaged on the Illumina iScan+.

### Genotyping analysis

SNPs were included in the analysis of dual-strain infection if they had a valid call in over 90% of all of the samples included in the study. At least 4 negative control samples were included on each plate. Clinical samples that did not have resolved calls in 9 or more SNPs were considered failed specimens and were excluded from the analysis (S1 Fig).

For individuals who had ≥5 HSV-2 mismatches between paired samples based on the array, we confirmed that the samples were from the same participant using the Investigator DIPplex Kit (Qiagen), which performs multiplex amplification of 30 polymorphic human INDELs and has been validated for human identification [28], according to manufacturer’s instructions. If samples could not be confirmed to be from the same person with this test, they were excluded from the analysis (S1 Fig).

### HTS

For the subset of individuals who met our criteria for dual-strain infection by the array-based genotyping assay, HTS of HSV-2 genomes was performed using the Illumina platform. DNA was fragmented using the Kapa HyperPlus Kit, and enrichment was performed using a custom IDT xGen oligonucleotide panel tiling the HSV-2 HG52 reference genome (NC_001798). Enriched pools of dual-indexed libraries were sequenced on an Illumina MiSeq using 2 × 300-bp sequencing runs. Reads were processed as previously described [17]. Briefly, raw reads were adapter- and quality-trimmed using BBDuk from the BBMap package version 36 [29] and de novo assembled using SPAdes version 3.9 [30]. Assembled scaffolds were mapped to the HSV-2 SD90 reference genome using Mugsy [31], gaps between scaffolds were filled with mapped reads, and a consensus sequence was constructed from the final merged alignment using a custom R script [32]. Allele frequencies were computed using LoFreq [33].

### Dual-strain infection prevalence sampling adjustment

We conducted a set of simulations to assess our ability to detect dual-strain infection with only 2 samples per person. True numbers of infecting strains were simulated for hypothetical people using a zero-truncated Poisson distribution indexed by λ; λ is a parameter that increases with the average number of infecting strains. We then randomly selected a single strain from each person, twice, to mimic the clinical experiment (code available online at https://github.com/dbrvs/goldengate_model). The resulting simulated data were compared with the experimental data to determine the true dual-strain infection frequency that best described the observed frequency. The sampling-adjusted estimate of dual-strain infection is subject to these assumptions: (1) accuracy of the zero-truncated Poisson distribution, which is akin to assuming a relatively even risk profile across populations, and (2) equal relative abundance, meaning that all infecting strains are detectable at the same frequency. Departures from the second assumption would inflate the adjusted dual-strain frequency further.

### Statistical analysis

Bayesian probabilities were computed to determine whether the selected SNPs could identify dual-strain infection. Bayes’s rule was used to estimate correct identification of identical/nonidentical sequences, with the following notation and equations. Let M indicate match at all SNPs (~M = mismatch) and I indicate that 2 sequences are truly identical at all loci (~I = not identical). Using standard Bayesian conditional probabilities for computing positive predictive and negative predictive values from prevalence of dual-strain infection p(~I), sensitivity of SNPs for dual-strain infection p(~M|~I), and specificity p(M|I), we compute the probability of correctly identifying identical sequences from matching SNPs p(I|M) and the probability of correctly identifying nonidentical sequences from mismatched SNPs p(~I|~M) using the following equations.

p(I|M)=p(M|I)*p(I)/[p(M|I)*p(I)+p(M|~I)*p(~I)]

p(~I|~M)=p(~M|~I)*p(~I)/[p(~M|~I)*p(~I)+p(~M|I)*p(I)]

The estimate for dual-strain infection was based on the number of pairs that met criteria for dual-strain infection divided by the total sample. The confidence interval (CI) for this estimate comes from the standard formula for variability of a binomial proportion.

Phylogenetic trees were created using the concatenated U_L__U_S_ regions, and maximum likelihood with bootstrapping (1,000 replicates) was used to compute support values as previously described [17].

Although we initially hypothesized 10% prevalence of dual-strain infection and planned to include 600 participants in the study, with a dual-strain infection prevalence of 5% and 400 participants, we had 89% power to detect a 3-fold increased risk of dual-strain infection in women and 81% power to detect a 3-fold increased risk of HSV-2 dual-strain infection based on HIV status and continent of origin. Poisson regression was used to assess potential univariable and multivariable associations of participant characteristics with dual-strain infection. Terms for interactions between HIV status and continent and HIV status and sex were tested. The initial multivariable model included sex, age, continent, lifetime number of sexual partners, HIV status, type of sexual partnership, and time between sample collections, with variables removed through backwards elimination. In response to requests from an anonymous reviewer, we also stratified the multivariable model by HIV status and performed a sensitivity analysis including 8 pairs of samples that were excluded due to failure of the assay to confirm that the samples were from the same person. *p*-Values ≤ 0.05 were considered statistically significant. This study is reported as per the Strengthening the Reporting of Observational Studies in Epidemiology (STROBE) guidelines (S1 Checklist).

## Results

### Samples to determine prevalence of dual-strain infection

Overall, 1,152 samples were selected for genotyping, including 1,093 clinical samples from 537 people, with 59 negative controls (S1 Fig). Of the clinical samples, 918 specimens from 459 people met the criteria of having valid calls at a minimum of 90% of HSV-2 loci in the array and having a paired specimen; 8 pairs that appeared superinfected were removed from the analysis because they could not be confirmed to be from the same person (S1 Fig). Of the 918 swabs, 638 (69.5%) were collected on days on which a lesion was present. The remainder of swabs were collected during asymptomatic shedding episodes. The baseline characteristics of the selected group are shown in Table 1; 213 (46%) were men, with a median age of 34 years (IQR: 27–44). Overall, 272 (59%) were from the US, 59 (13%) were from Peru, 128 (28%) were from 1 of 8 African countries (including Botswana, Cameroon, Kenya, South Africa, Tanzania, Uganda, Zambia, and Zimbabwe). Infection with HIV was present in 130 (28%) people. The median lifetime number of sexual partners was 11 (IQR: 3–32). Of 438 people who reported type of sexual partnership, 382 (65%) identified as heterosexual, and 155 (35%) were men who had sex with men.

**Table 1:** Baseline characteristics of the study population, stratified by the presence of dual-strain infection.

Characteristic	Dual-strain infection (*n* = 17)	Single-strain infection (*n* = 442)	All (*n* = 459)
**Male**	6 (35%)	207 (47%)	213 (46%)
**Age, median (IQR)**	30 (27–40)	34 (27–44)	34 (27–44)
**Continent**			
North America (US)	2 (12%)	270 (61%)	272 (59%)
South America (Peru)	3 (18%)	56 (13%)	59 (13%)
Africa*	12 (71%)	116 (26%)	128 (28%)
**Lifetime number of sexual partners, median (IQR)**	6 (1–25)	11 (3–34)	11 (3–32)
**HIV seropositive**	12 (71%)	118 (27%)	130 (28%)
**Sexual partnership**			
Heterosexual	11 (79%)	272 (61%)	283 (65%)
Men who have sex with men	3 (21%)	152 (36%)	155 (35%)
Not asked or not provided	3	18	21
**Months between samples, median (IQR)**	5 (3–11)	5 (2–11)	5 (2–11)

Data are given as number (percent) unless otherwise indicated.

*Botswana, Cameroon, Kenya, South Africa, Tanzania, Uganda, Zambia, or Zimbabwe.

### Identification of SNPs for genotyping platform and validation

To obtain information for SNP selection, we sequenced HSV-2 DNA collected from genital swabs from 38 people in the US, Peru, and several African nations as described above [17]. From these sequences we identified 456 population-prevalent SNPs and prioritized SNPs that could best distinguish strains from one another for creation of a high-throughput genotyping assay (S2 Fig). The SNPs selected for development of the custom genotyping assay are shown in S1 Table. Eleven (11.5%) of the 96 loci selected failed the prespecified quality criteria and were subsequently excluded from analysis of dual-strain infection, leaving 85 loci for analysis. We estimated that based on the prevalences of these 85 SNPs and assuming a dual-strain infection frequency of 10% and a call error rate of 1%, there was a >99% probability of correcting calling identical sequences and a 92% probability of correctly identifying nonidentical sequences.

To confirm that the array-based genotyping assay correctly identified HSV-2 sequences, we determined whether the genotype matched the result from HTS at each locus in 5 samples that underwent both HTS and array-based genotyping. Among these 5 samples, there were matches at all positions with valid results for both methods (a total of 449 loci) and no mismatches.

To confirm that these selected loci could distinguish between strains, we used 70 HSV-2 full-length or partial genetically nonidentical HSV-2 sequences available in GenBank [17,34] and found that these 85 loci could distinguish all but 2 pairs of strains (out of 2,415 combinations) (99.9% specificity). We evaluated the number of mismatches among sequences collected from different people, and found that most samples had at least 5 mismatches (1,905 [99.2%] of 1,920 observations) and that the median number of mismatches between unrelated pairs was 23 (IQR: 19–27). Because fewer than 1% of samples had <5 mismatches, and due to concern that paired samples with fewer than 5 mismatches could have represented within-host evolution or sequencing error, at least 5 mismatches were required between pairs to diagnose dual-strain infection.

### Determination of dual-strain infection based on number of SNP mismatches between paired specimens

**Fig 1:**
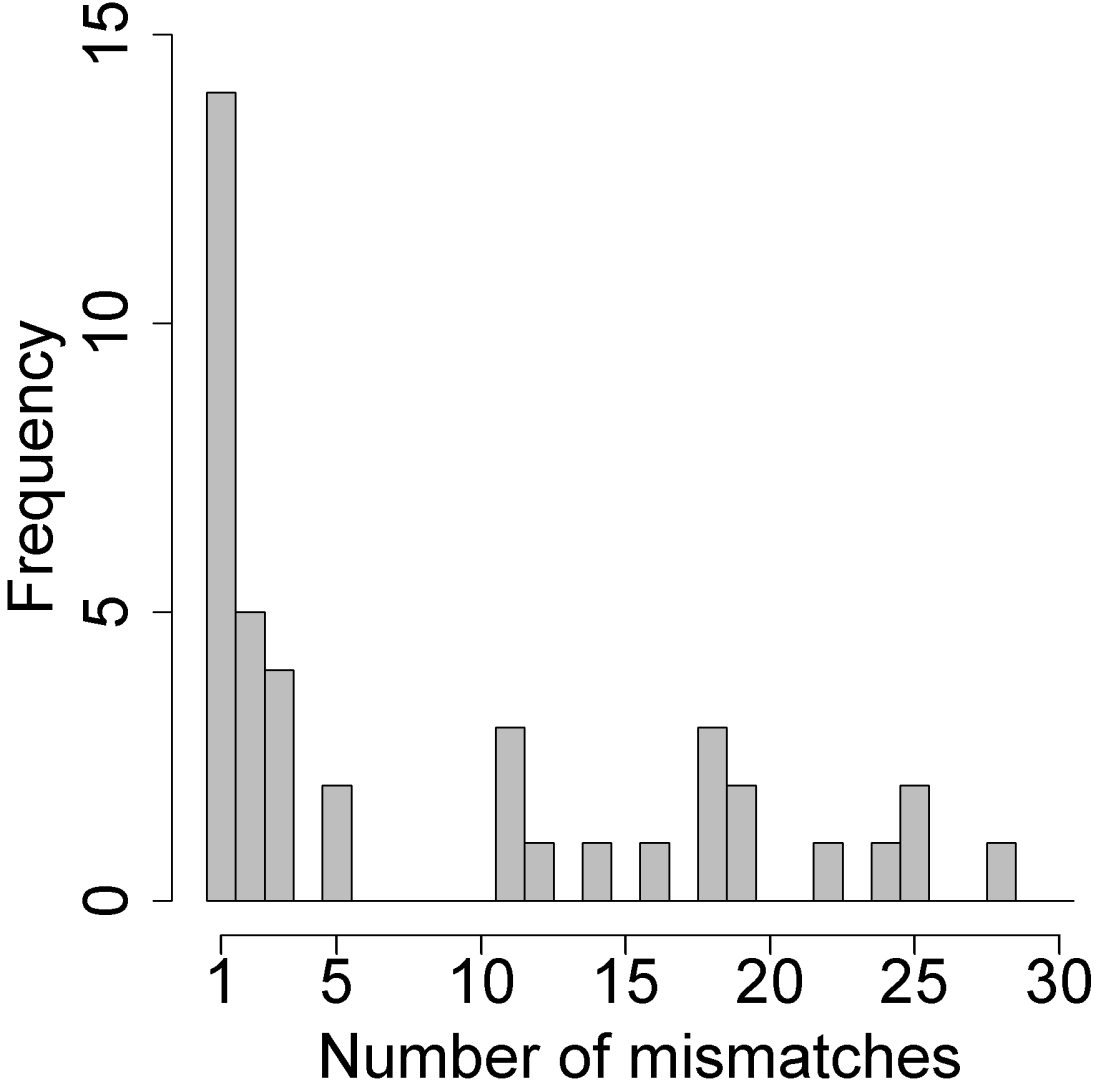
Histogram of the number of mismatches using a custom genotyping assay for each of the 41 pairs with any mismatches. Pairs with ≥5 mismatched SNPs were considered superinfected; those with 1–3 mismatched SNPs were not subject to confirmation by high-throughput sequencing.

Of the 459 participants with paired samples, 418 (91%) participants had identical SNPs in the paired specimens. The remaining 41 pairs were mismatched at a median of 3 SNPs (IQR: 1–28). As shown in Fig 1, 14 (34%) differed at only 1 locus. Eighteen pairs had ≥5 mismatches and met our prespecified criteria for dual-strain infection. Among these 18 pairs, the median number of mismatches was 18 (IQR: 11–21). For each pair that appeared to have mismatched HSV-2 strains, we confirmed that the specimens came from the same person using a multiplex amplification kit developed for human identification.

### Confirmation of dual-strain infection using HTS with oligonucleotide enrichment

**Fig 2:**
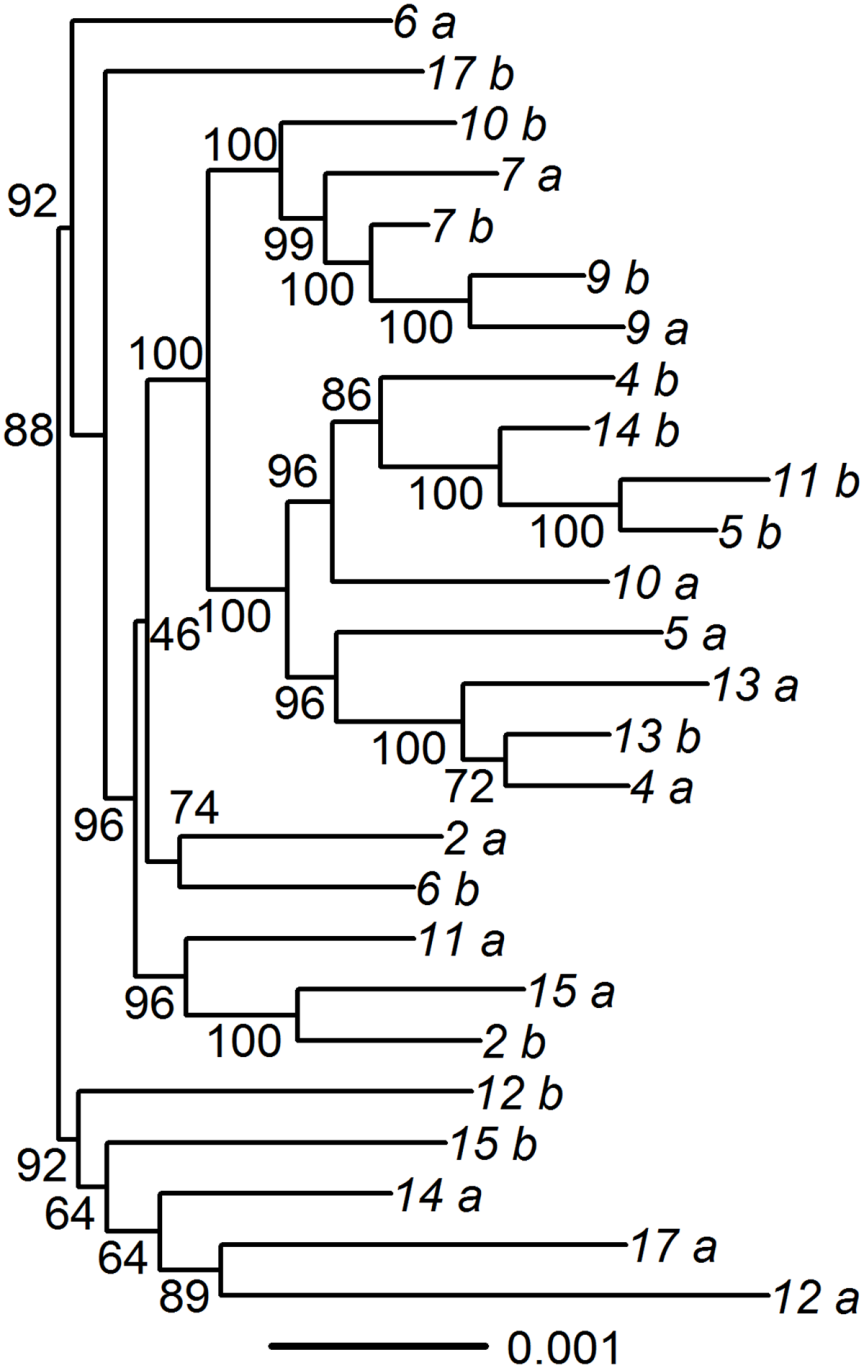
Phylogenic tree of paired specimens that underwent oligonucleotide enrichment and high-throughput sequencing. The concatenated U_L__U_S_ regions were used to create the phylogenetic tree, using maximum likelihood. Bootstrapping with 1,000 replicates was used to compute support values. Paired specimens are indicated by number with “a” and “b.” Pair 3, which was found to be incorrectly identified as superinfected using the array, was excluded from the tree.

For 18 pairs that appeared to have different HSV-2 strains present in the 2 samples, we performed DNA oligonucleotide enrichment followed by HTS (S2 Table). High-quality sequences were obtained from 14 pairs; the remaining 4 pairs were not able to be sequenced but were retained in the analysis as superinfected pairs. We also confirmed mismatches at array sites using HTS data by determining whether the majority base at each array site for paired samples was a match (S3 Fig). For one pair (Pair 3) we found that the SNP mismatches identified by the array were not found in the full genome sequence, suggesting that one of the samples in this pair was contaminated or mislabeled, and therefore we removed this pair from the analysis of dual-strain infection. For the remaining 13 pairs, we observed a median of 274 mismatched SNPs in the genomic sequence (range 129–413). Phylogenetic analysis revealed that most paired but unique specimens did not cluster together on the tree, supporting that the sequences represent 2 different strains rather than within-host evolution (Fig 2). The median Tamura–Nei distance between the pairs was 0.0023 (IQR: 0.0020–0.0025). We explored whether minor variants representing the strain found at one time point could be found within the dominant strain of the other time point. We found minor alleles at frequencies > 2% at array sites in 10 out of 14 pairs, but we were unable to consistently detect signatures of the other strain at these sites.

### Estimated prevalence of and risk factors for dual-strain genital HSV-2 infection

Overall, using the genotyping assay we identified infection with 2 different strains in 17 (3.7%) of 459 people (prevalence = 3.7%, 95% CI = 2.0%–5.4%), with dual-strain infection confirmed using HTS in 13 persons. Using simulated data that account for the possibility of detecting a single strain by chance even when multiple infecting strains are present, we inferred the underlying prevalence of dual-strain infection to be ~7% (S4 Fig).

In univariable analysis, we found that sex, age, lifetime number of sexual partners, and type of sexual partnership (heterosexual versus men who have sex with men) were not associated with dual-strain infection (Table 2). However, the univariable analysis revealed that people from Peru and from Africa had a 6.9-fold (95% CI = 1.2–40.3) and 12.8-fold (95% CI = 2.9–55.7) increased risk of dual-strain infection, respectively, compared to people from the US. People with HIV infection had a 6.1-fold increased risk of dual-strain infection (95% CI = 2.2–17.0) compared with HIV-negative people. In a multivariable model including continent of origin and HIV serostatus, collection from Africa (risk ratio [RR] = 9.20, 95% CI = 2.05–41.32) and HIV seropositivity (RR = 4.06, 95% CI = 1.42–11.56) remained significantly associated with HSV-2 dual-strain infection. In the multivariable analysis, we tested the interaction between HIV status and continent and did not find a significant interaction. However, to exclude the possibility that the higher frequency of dual-strain infection in Africa could be due to residual confounding by HIV infection, we also stratified the multivariable analysis by persons with and without HIV infection; the RR for being from Africa was 8.57 among persons without HIV infection (*p* = 0.06) and 9.52 among persons with HIV infection (*p* = 0.003). In a sensitivity analysis including 8 pairs that appeared superinfected but were excluded due to inability to confirm they were from the same person, nearly identical findings were demonstrated in the multivariable model, although the strength of the association was attenuated (collection from Africa: RR = 4.38, 95% CI = 1.69–11.36; HIV seropositivity: RR = 2.81, 95% CI = 1.25–6.34) (S3 Table).

**Table 2:** Risk factors for dual-strain infection, analyzed by Poisson regression.

Characteristic	Univariable analysis		Multivariable analysis*	
	Risk ratio (95% CI)	*p*-Value	Risk ratio (95% CI)	*p*-Value
**Male**	0.63 (0.24–1.68)	0.36	—	—
**Age in decades**	0.74 (0.47–1.18)	0.20	—	—
**Continent**				
North America (US)	Ref	Ref	Ref	Ref
South America (Peru)	6.92 (1.19–40.26)	0.032	5.10 (0.86–30.47)	0.074
Africa*	12.75 (2.92–55.66)	<0.001	9.20 (2.05–41.32)	0.004
**Lifetime number of sexual partners, each additional 10**	0.87 (0.64–1.19)	0.39	—	—
**HIV seropositive**	6.07 (2.18–16.94)	<0.001	4.06 (1.42–11.56)	0.009
**Men who have sex with men**	0.50 (0.14–1.75)	0.28	—	—
**At least 3 years between samples**	2.42 (0.57–10.31)	0.23	—	—

*In multivariable analysis, there was no significant interaction between HIV status and continent or HIV status and sex in predicting prevalence of dual-strain infection.

## Discussion

In this report, we systematically studied the prevalence of HSV-2 dual-strain infection using paired genital swab samples collected at 2 different time points from 459 people in 3 continents. Among 18 pairs of samples that appeared to have different strains at the 2 time points by genotyping, we definitively confirmed that 2 different strains were shed at different time points in 13 people using whole genome sequencing, and were unable to perform confirmatory sequencing in 4 pairs. These results revealed that the prevalence of HSV-2 dual-strain infection was 3.7%, with sampling-adjusted estimates indicating that the true prevalence may be 7%. In multivariable analysis, dual-strain infection was associated with people who were from Africa and people with HIV infection. The finding that HSV-2 dual-strain infection was relatively rare in this sample may indicate that the natural immune response is usually sufficient to protect against infection with a second strain of HSV-2. These data suggest that a vaccine format that elicits a breadth and level of specific immunity that meets, or exceeds, that achieved by natural genital HSV-2 infection may be efficacious in preventing infection with wild-type virus.

We demonstrated that population-prevalent SNPs identified using HTS could be used to identify dual-strain infection using a custom genotyping tool. The ability to use genomic sequences to determine which high-yield SNPs to use for genotyping is just one application of HTS, which has the potential to significantly advance our understanding of the pathogenesis of HSV-2 infection. In this work, we built a genotyping platform and validated it using HTS. While rapid genotyping methods are in a state of flux characterized by improvements in cost, throughput, and specimen requirement, it is important to validate each candidate method. Although the SNPs selected for genotyping in this analysis might not as reliably differentiate strains from people from other geographic areas, the rapidly growing database of full-length and partial HSV-2 genomes will facilitate future genomic analyses of this type [17,34–38].

In this study, we were not able to determine whether people with dual-strain infection acquired the 2 strains at the time of the initial HSV-2 acquisition, or whether they acquired the strains sequentially. However, based on HTS studies performed to date, both in this sample and prior studies, simultaneous shedding of 2 strains was detected in only 1 (1.8%) of 55 people studied. The low frequency of dual-strain shedding within individuals detected to date suggests to us that acquisition of 2 strains simultaneously from 1 exposure would be less likely than acquisition of 1 strain followed by a second strain at the time of a second exposure. Only a prospective study of a cohort enrolled during primary HSV-2 infection could adequately address this issue. Studies of full-length HSV-1 and HSV-2 sequences have revealed evidence for pervasive HSV-2 × HSV-2 and HSV-1 × HSV-2 inter-strain recombination, implying dual-strain infection of individuals, and indeed of single cells, with 2 strains of HSV-2 or simultaneous infection with HSV-1 and HSV-2 [37,39].

Dual-strain infection has been explored with other herpesviruses using both classic and newer sequencing-based methods to determine the presence of multiple strains. Infection with a second, heterologous cytomegalovirus (CMV) strain has been demonstrated in nearly 1/3 of healthy, young, previously pregnant US women followed over a 3-year period, based on development of new antibody formation against a polymorphic epitope [40], but the study did not identify risk factors for infection with a second strain. In addition, data consistent with mixed CMV infection were found in nearly half of 28 newborns with congenital CMV infections [41]. Whole genome sequencing of longitudinally collected specimens from congenitally CMV-infected infants has demonstrated mixed-strain CMV infection in 1/3 of patients and suggests that maternal reinfection occurs frequently [42,43]. Infection with 2 strains of varicella zoster virus has also been demonstrated in case reports [44]. The prevalence of HSV-2 dual-strain infection found in the present analysis, 3.7%, was much lower than the prevalence of mixed-strain CMV infection in healthy US women [40]. The much longer follow-up period in the CMV study in young women suggests that the risk of superinfection may be cumulative over time.

To our knowledge, this study is the largest investigation of HSV-2 dual-strain infection to date, and we utilized a novel genotyping strategy to detect dual-strain infection. Initial studies of HSV-2 genotyping using restriction length polymorphisms to genotype specimens indicated that dual-strain infection was possible [13–15]. Using PCR-based genotyping methods based on DNA repeats, Roest et al. identified evidence of dual-strain infection in 11 of 11 people with HIV infection and 1 of 8 people without HIV infection [16]. Burrel et al. characterized microsatellite repeats within the HSV-2 genome to develop a PCR-based genotyping assay based on polymorphisms within 12 microsatellite regions and were able to differentiate 56 strains from Western Europe and West Africa [45]. In a previous study in which we performed HTS on paired samples from 8 people, we found HSV-2 dual-strain infection in 2 people with HIV infection [17]. However, with such small numbers of participants, it is difficult to make definitive conclusions about risk factors associated with dual-strain infection.

In this large study population selected from longitudinal natural history studies and international clinical trials we found that HIV infection is a significant risk factor for HSV-2 dual-strain infection, with 4.0-fold increased risk compared to HIV-seronegative people. As the participants with HIV infection in this sample had CD4 counts > 350 cells/mm^3^ at enrollment into the studies, it is unlikely that severe immunocompromise was responsible for the increased risk of dual-strain infection. HSV-2 is a prevalent infection in HIV-seropositive people, and the increased risk of HSV-2 dual-strain infection in this population may be a result of increased risk of sexual exposure to heterologous HSV-2 strains [22,24]. We were not able to detect an independent contribution of lifetime number of sexual partners. Prospective studies of sexually active people with and without HIV infection are required to determine if HIV infection in the absence of overt immune compromise, or specific sexual practices, are independent risk factors for HSV-2 dual-strain infection.

This study had several important limitations. Due to the cross-sectional design of this study, we were able to determine the prevalence, but not the incidence, of dual-strain infection; the incidence would provide a better estimate of the effectiveness of naturally occurring HSV-2 immunity against reinfection with a second strain of virus. Importantly, prospective cohort data would allow researchers to differentiate between acquisition of 2 strains simultaneously during primary infection versus sequential infection of a second strain after new exposure. We did not have information about all sexual partners in the interim between sample collections, and therefore whether people were at risk for acquiring dual-strain infection during the follow-up period is unknown. In prospective cohorts, associations between incidence of HSV-2 dual-strain infection and risks of exposure, including number of sexual partners and sexual encounters, can be ascertained. In addition, we were not able to determine whether HSV-2 dual-strain infection results in any difference in clinical HSV-2 presentation, such as increased rates of mucosal shedding or genital ulcer disease, but this would be an area of interest for future studies. Due to many specimens failing our strict quality criteria for the genotyping assay, we had a smaller sample size in the analysis than originally planned. There may have been small differences in the risk of dual-strain infection between groups, for instance women and men, that we were underpowered to detect. Due to the assay design, we were unable to test samples with <5 log_10_ copies HSV DNA/ml; it is possible that additional strains would be shed only at lower quantities, resulting in an underestimate of dual-strain infection. Dual-strain infection prevalence may differ in populations from different parts of the world or in populations with high rates of sexual exposure to HSV-2 infection. The increased risk shown in the African population may be a reflection of higher HSV-2 seroprevalence in this population, and therefore higher risk for exposure to different strains [21], or may be because of bias due to defining dual-strain infection based on array-based SNP genetic distance: HSV strains isolated from African populations show a higher amount of genetic distance from each other; thus, we may be undercounting dual-strain infection in other geographical areas [46]. The sampling-adjusted estimate of dual-strain infection assumes that the zero-truncated Poisson distribution is accurate and that strains are detectable at the same frequency. To evaluate departures from the zero-truncated Poisson assumption, we are exploring the impact of varying HSV-2 acquisition risk within the study sample.

In summary, we measured the frequency of HSV-2 dual-strain infection in a large sample of people from around the world and showed that infection with more than 1 strain was rare in this sample. Understanding the frequency of and risk factors for HSV-2 dual-strain infection is important for the future design of prophylactic vaccine studies. Based on these results, we hypothesize that generation of a broad immune response to HSV-2 infection, similar to naturally induced immunity, could be protective against infection with a second strain. Future studies using genomics and genotyping may lead to further insights into HSV-2 biology that bring us closer to the development of a successful prophylactic HSV-2 vaccine.

## Supporting information

S1 Checklist STROBE checklist indicating adherence to the STROBE guidelines. (DOC)

S1 Data Demographic information and sexual history information for each participant. People with dual-strain infection are indicated by “1” in column K; those without dual-strain infection detected are as indicated by “0.” The number of mismatches detected by genotyping assay is indicated in column L.
(CSV)

S2 Data Data used to make S3 Fig. (CSV)

S1 Fig Flow chart of participant selection. (TIFF)

S2 Fig Heat map of population-prevalent SNPs used to select SNPs for genotyping assay. “hclust” was used to rank the population-prevalent SNPs that would best differentiate between strains. Each row is an SNP, and each column represents the HSV-2 sequence from a single person. The heat map sorts the SNPs left to right by sequence cluster and from top to bottom by similarity within cluster. Blue indicates the same SNP, whereas yellow indicates the alternate genotype.
(TIFF)

S3 Fig For each pair of samples, we validated custom array mismatches using HTS by comparing the majority base at each site for the paired samples. The outlier with 22 mismatches by the custom array (GG) and 1 mismatch by sequence is Pair 3, which was excluded from the analysis of superinfected pairs.
(TIF)

S4 Fig Dual-strain infection is underestimated when the observed prevalence is used. (A) The average distance between simulated and observed data in an experimental trial with 20 replicate simulations. The error is minimized when parameter λ is 0.15. (B) The observed data compared directly with the best-fit simulation. About 3% of people observed to have only 1 strain are anticipated to have at least 2 strains present. The true prevalence of dual-strain infection is the sum of all the probabilities with *n* > 1. Thus, for the best-fit parameter, the dual-strain infection prevalence is 7.2% instead of 3.7%.
(TIF)

S1 Table Oligonucleotides used to create the custom HSV-2 genotyping assay. The nucleotide in brackets represents the SNP of interest.
(DOCX)

S2 Table Baseline characteristics and GenBank number for samples that underwent HTS. (DOCX)

S3 Table Sensitivity analysis of risk factors for dual-strain infection, including 8 pairs that were excluded from the analysis due to inability to confirm the 2 samples were from the same person. (DOCX)

S1 Text Original power calculations and data analysis plan. (DOC)

## Abbreviations

CI: confidence interval
CMV: cytomegalovirus
HSV: herpes simplex virus
HSV-2: herpes simplex virus type 2
HTS: high-throughput sequencing
RR: risk ratio
SNP: single nucleotide polymorphism
UW-VRC: University of Washington Virology Research Clinic
