# Supplementary figures and images for: Dual-strain genital herpes simplex virus type 2 (HSV-2) infection in the US, Peru, and 8 countries in sub-Saharan Africa: A nested cross-sectional viral genotyping study
Source: PLoS Med. 2017 Dec 27;14(12):e1002475. doi: 10.1371/journal.pmed.1002475 (PMC5744910; doi:10.1371/journal.pmed.1002475)

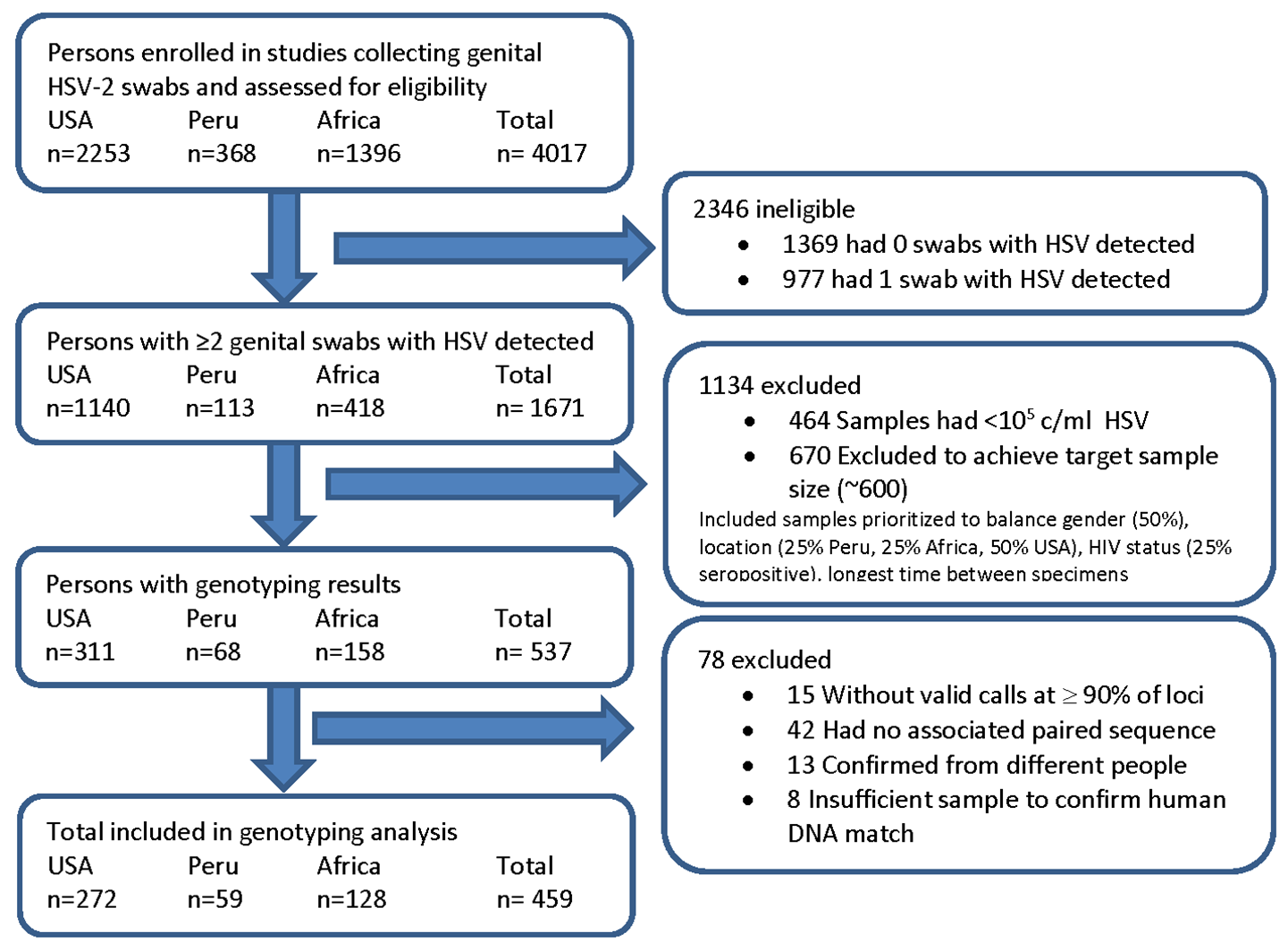

Supplement: S1 Fig — (TIFF) [file pmed.1002475.s004.tiff]

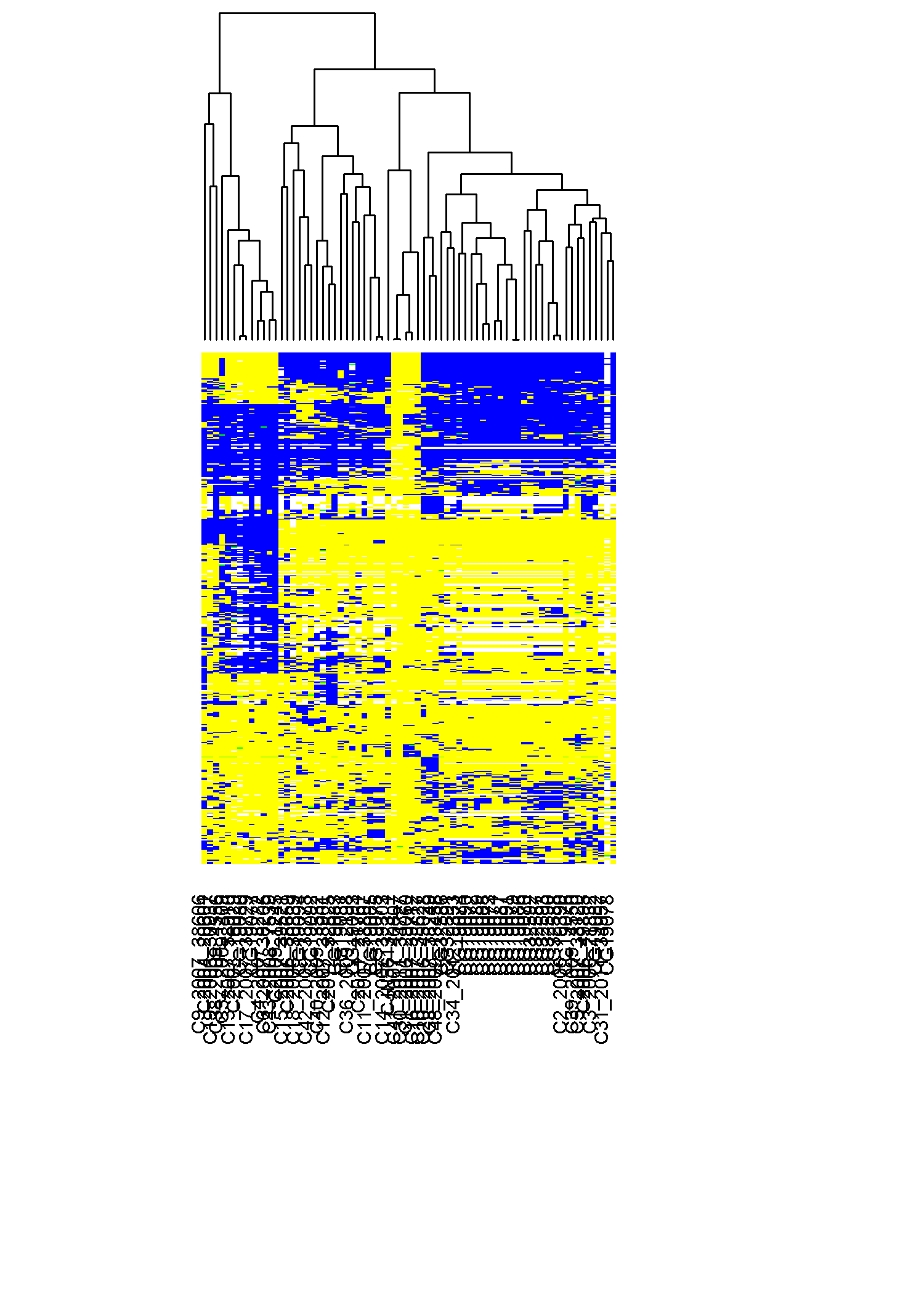

Supplement: S2 Fig — “hclust” was used to rank the population-prevalent SNPs that would best differentiate between strains. Each row is an SNP, and each column represents the HSV-2 sequence from a single person. The heat map sorts the SNPs left to right by sequence cluster and from top to bottom by similarity within cluster. Blue indicates the same SNP, whereas yellow indicates the alternate genotype. (TIFF) [file pmed.1002475.s005.tiff]

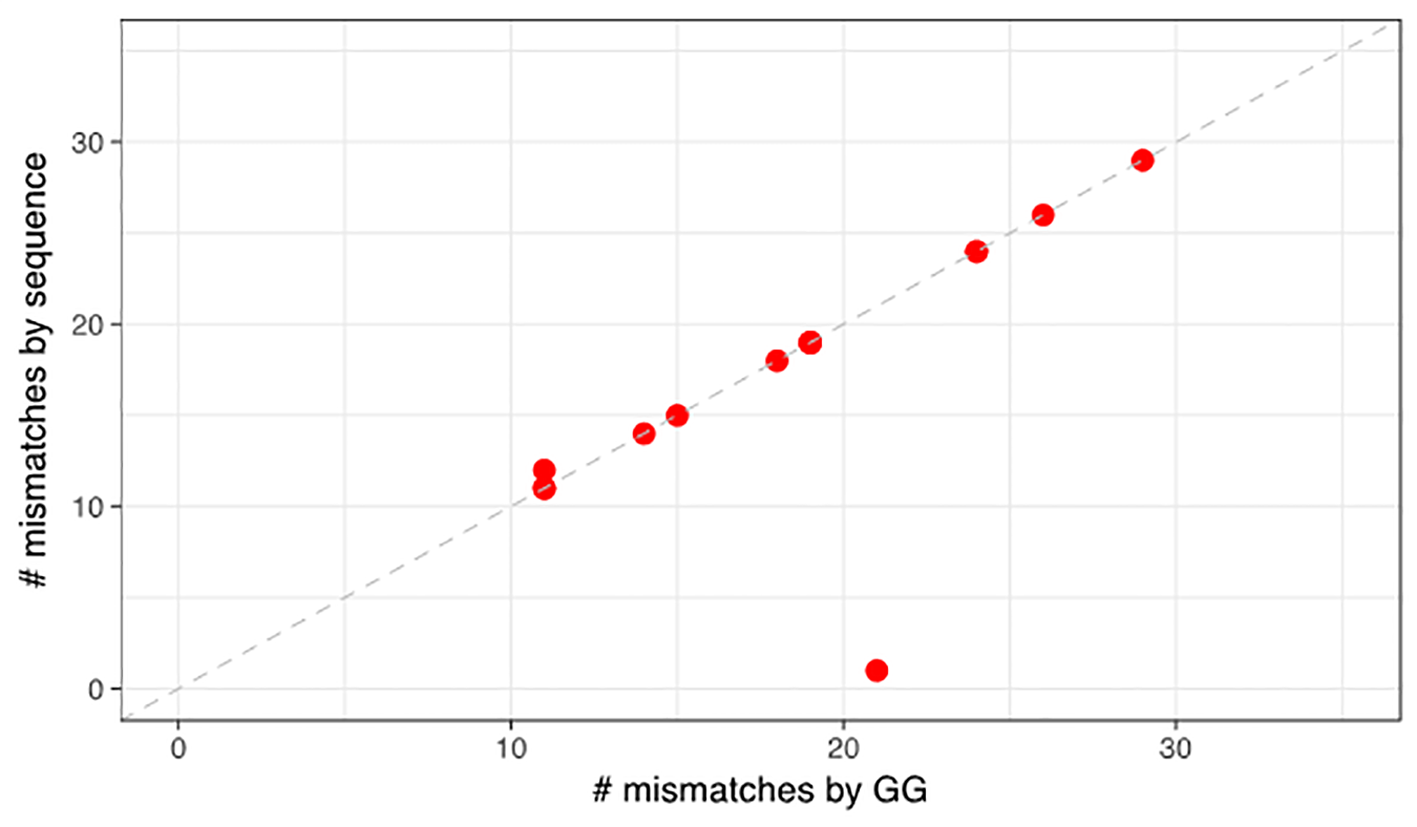

Supplement: S3 Fig — The outlier with 22 mismatches by the custom array (GG) and 1 mismatch by sequence is Pair 3, which was excluded from the analysis of superinfected pairs. (TIF) [file pmed.1002475.s006.tif]

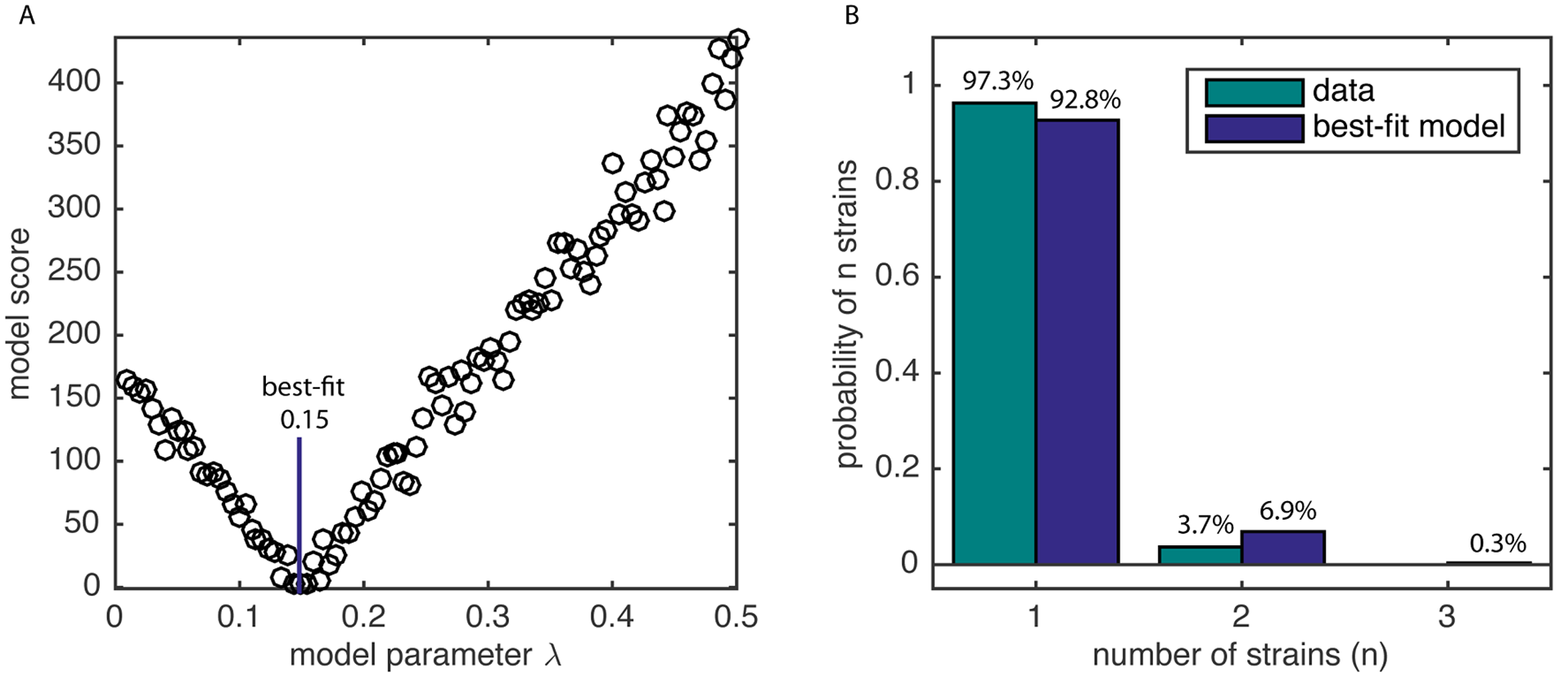

Supplement: S4 Fig — (A) The average distance between simulated and observed data in an experimental trial with 20 replicate simulations. The error is minimized when parameter λ is 0.15. (B) The observed data compared directly with the best-fit simulation. About 3% of people observed to have only 1 strain are anticipated to have at least 2 strains present. The true prevalence of dual-strain infection is the sum of all the probabilities with n > 1. Thus, for the best-fit parameter, the dual-strain infection prevalence is 7.2% instead of 3.7%. (TIF) [file pmed.1002475.s007.tif]
