# Supplementary material for: Dual-strain genital herpes simplex virus type 2 (HSV-2) infection in the US, Peru, and 8 countries in sub-Saharan Africa: A nested cross-sectional viral genotyping study
Source: PLoS Med. 2017 Dec 27;14(12):e1002475. doi: 10.1371/journal.pmed.1002475 (PMC5744910; doi:10.1371/journal.pmed.1002475)
Supplement: S1 Table — The nucleotide in brackets represents the SNP of interest. (DOCX) [file pmed.1002475.s008.docx]

| S1 Table. Oligonucleotides used to create the custom HSV-2 genotyping assay. The nucleotide in brackets represents the SNP of interest | | |
| --- | --- | --- |
|  |  |  |
| SNP | Oligo | Position: JN561323 |
| 1* | CTATCACTGCCCGGGGTTGGACACGTTTTTGTGGGATAGGCACGCCCAGAGGGCGTATCT[T/G]GTTAACCCCTTTCTCTTTGCGGCGGGATTTTTGGAGGACTTGAGTCACTCTGTGTTTCCGG | 9741 |
| 2 | GTTTAGCGTGCGTGCGGATGTGCCGGTGCCTCCGAGTCTACGGAACGTGCTGGCGGCGGT[A/T]AAAAATTGTTACCCCGACGCGCGCATGAGCGGCCGCGGCTGCCTGGAAAAGTGGGCTCGCG | 10639 |
| 3 | CTCGCCGCGAATCGCTACCTCGAAACCCGGGACATTATGCCGATCGACTGGTCGGTATAA[A/G]ATGCCGACATCCGGGGTCTTGATTTACGAGGGGGCAATTAATAAAGACTGTTGATGGTTAA | 11015 |
| 4 | TGGGGCTGATCTGAATTTCCCGCAGAACCTCGACCAGTAGGTCTGTTGTGTTTGCTGGGA[A/C]CTCGCCCGCCGTTGGGGATACGGGGGCGGGGGGTGTGGTCGGGCGGACGTCCAGGGGTGCG | 12065 |
| 5 | CTCCCGGAGCGGGTTTAGATTCATGTGCAGGAACTCGGATGAGGTGGTGCGGGACATGGC[T/G]ACGTACGCGCTGTTTAGGCGCAGGTTTCCGGGCGTGAAGCATATGGCGACCTTGTCCAGAC | 12780 |
| 6 | CAAACTCACTGATGTTGGTATTGACGACAGACATGAAGCCGTGCTGGTCCCGCAGGACGA[T/C]GTAGGGCAGGGGGGACTCCTCCAAGAACTCGGCCACGCCGGCCGTCGCGTGCCGCCGCCGC | 13001 |
| 7* | GTATCGCCCCAGAAAGGCCCTCTTCAGGAGCCCAAACTGGGCGTGGACGGCCGCGGTGGT[T/C]TCAGGCTCTTCGAGGGCGTAGTGGCAGTAGAACACGTCCAGCTGCTGTTCGTCCAGCCCGG | 13320 |
| 8 | GTTGAGGACGTACGTGATGTCGTTCCGGGCCACGACTAGCTGTTGCTTGCTGTGCACCTC[A/G]CAGCGCACGTGCCCCGCGTCCTGGTCCTGACTCTGGGAGTAGTTGGTGATGCGACTGGCGT | 13860 |
| 9 | CAGCGTGGGTTGCTGCGTGAGCCGTCGATACTCGTCAAACTCTTTGACCGACACAAACGT[A/G]AGCACGGGGAGGGTAAACACAACAAACTCCCCCTCGCGAGTCACCTTTAGGTAGGCGTGGA | 14007 |
| 10 | GCGGTTTACCGCGACCAGCGTCCTGCGCGTTGACGTCACCCCCAGGGGGCGGTTGCGGTT[T/C]GTTCTGGACGGGAGTTCCGACGACGCGTACGTGGCGTCGGAGGATTACTTTAAGCGCTGCG | 17515 |
| 11 | GGGGCGGTTGCGGTTCGTTCTGGACGGGAGTTCCGACGACGCGTACGTGGCGTCGGAGGA[T/C]TACTTTAAGCGCTGCGGGGACCAGCCGACGTATCGCGGTTTTGCGGTCGTCGTCCTCACGG | 17560 |
| 12 | CGTCCCCCGCACGGACTGTGTGGCCTATAACCCGGCCGGCGTCATGGGAAGCTGCTGGAA[A/T]TCCAAGGACCTGCGTTCGGCTCTGGTGTATTGGTGGCTTTCGGGGAGCCCCAAACGACGGA | 18097 |
| 13 | CGCTTTTCTATCGGTTTTGCTAACTCCGGAAAATAAACGTGTTTTTTATGGAACGTTCCC[T/C]ACCTGTCGTGTCATCTCTCGGGGGATGGTGGTGGGCCTGTGTGTGTGTCTTGTGCACCGAA | 18225 |
| 14 | GTGGTGGAAAGACATGATAGAGGGAACAAAGAAATAGAAGAAAACCACAACCGGCGCGTG[T/C]CAGTAAATACGGACGCGCGCACACGCGGGGGGTAAGTTGGAGCACGGGGCCCCGGTTTATT | 18375 |
| 15 | GTCCGCGAAGGCGCCCTCCTTCCTGGTCGGCGGAAACGCCAGGGTGGTGTATTCGCGCGC[A/G]AAACGCGCGGTCCTCGTCGTGATGGTGACGGCGAGCGAGGCGGAGGACGCGCACTGGGGGC | 20080 |
| 16 | CGCGCGCAGCTTGAAGCGCGCGCCCGCAAACTCCCGCTTATGGGCCATCAGCAGCGCGTA[T/C]AGCTGTCTGTGCGTCCGGCAGGCGCTGTGGTCGATGCGGTGGGCGTCCAGCAGCTCCACGA | 20954 |
| 17 | GTCGTAGTTGTTCAACAGGTTCGGGCCCACGCGATGAAGACTTTCCACCTGCACGATGAG[A/C]CGGTGGAAGGGGCGGTCGTTCATGATGTAATTGGTGGATGAGAAGTAGGTGACGAAGTCGG | 22856 |
| 18 | GCTAATCGCCGGAAACGTCTGCGCGTGGCTGTTGCAGATCACAGTCCTGCTGCTGGCCCA[T/C]CGCATCAGCCAGCTGGCCCACCTTATCTACGTCCTGCACTTTGCGTGCCTCGTGTATCTCG | 23723 |
| 19 | ACAACAAAAAATACCACACATACGACCAAATACGGACAATCATTTCTGTCTTTATTCGCT[A/G]TCAGAGAGTGGGGGCGTGAGCGTGGCAGGAGGGCGGGCCACGTCGGGGTCCCGCCGTCTGG | 24857 |
| 20 | CTGCTGCAGCGAGAACCCGAGCGGGGTGATAAAGCCGCGGATGTCGTGGGTGCGGCCGCC[A/G]CGAAGAGCGCACTCCCCCACGAGCAGGGTCGCGACGAGCTCCACGGCAAACCACTCTTTTT | 27914 |
| 21 | GCCGCGAAGAGCGCACTCCCCCACGAGCAGGGTCGCGACGAGCTCCACGGCAAACCACTC[T/C]TTTTCCCGGATGGTCTTCACGGCGAGCTTGTGTTCGCGAATCAACTGCACCTCGCCGTACC | 27971 |
| 22 | TTTACTCTGCTCGCGTCCATTGACGTCACCGTCAATCACCACTGCGATTGGACGGTTGGT[A/G]AGGCGCAGCGTGTCTCCGCTGGTGCTGTAGTAGTCAAACGCGTAGTGGGCGTCGGAGTCGG | 31710 |
| 23 | AACATCGGGAAAACGGATTCCCGCACGTGCGTCTTCCCAGATTCGACACACACACCCCCC[T/G]TCTCCTTAAATAAACACAAACCACACGCTCGTTGGTTGGTTAATGCCAGCGCTTTATTTAC | 35034 |
| 24 | CGTGCGTCTTCCCAGATTCGACACACACACCCCCCTTCTCCTTAAATAAACACAAACCAC[A/G]CGCTCGTTGGTTGGTTAATGCCAGCGCTTTATTTACGTCTTGTTTTTTTTGCGTTTCCTCC | 35059 |
| 25 | TCGCGTGACGCTCTGCATCTGCAGGAGCGCGTTCACGTATCCGTCCTGGGCGCTCAGCGC[C/G]AGCAGCCGGGGGATGAGCGTGAGGATGAGGGTGGTTCCTTCGGTTATGGAGTAGACCATGT | 35426 |
| 26 | GGTTCCTTGGGGGATTCGCAGGCTCCATCAAGCCGAGCTCGGGAAGGCCAAGCCCCTCCC[A/G]CACAACGCCTCACCGCCGGCGGACGCGACTAACAACCCACGGGCCGCCAAAACCCCAAGGG | 40673 |
| 27 | GCCAAGCCCCTCCCGCACAACGCCTCACCGCCGGCGGACGCGACTAACAACCCACGGGCC[A/G]CCAAAACCCCAAGGGGCAACCCGACCAACAACAGGCGAGGGGAGGAAAGGCGTAAAGGGGG | 40719 |
| 28 | AAAAATCAGACGTGCCGTACGAGGACAGCGAAAACTGTTCATCGAGCGGCAGTTCTCCGT[T/C]CTCCCCGCCACACGCGGCCTCGTCTACCAGCTCGCGATCCAACAAAGGAACATCATCCCGC | 41521 |
| 29* | TCTCCACAAAAATGGGACGCACGTTCGGACCACCCTAAGGATGCCCGCCAGGGCCGCGGT[A/G]ATCATAACGACCCCCAGCGCGGACGCGGCCAGAAACCCGGGGGCGATGGTGGCGATGGGCA | 44123 |
| 30 | GAGGTACACGTACCGACCCGGAGTCCGTAGCAGGCCCCTGGCGGCCAGCCAGGTCACGGA[T/C]GCGTTGTGCAGATGCGCGATGCTCAGGTTCGTCGTCGGATGCCTCGGTGTCCCCGCGGGCG | 45920 |
| 31 | GTGGGCCCCGCCTCGGGGAGCTCGGGCAGGCTCGCGTTCCGAGGCCGGCCGAGCAGATAG[C/G]TCTTTGGGATGTAAAGCAGCTGCCCGGGGTCCCGAGGAAACTCGGCCGTGGTGACCAACAC | 46236 |
| 32 | CGAACGCAGGCGCGTGCTGTTGGCCGGCGTGAGAAGCCATACCCGCTTCTACAAGGCGTT[T/C]GCCCGAGAGGTGCGGGAGTTCAACGCCACCAGGATTTGTGGAACGCTGCTGACGCTGATGA | 48060 |
| 33 | GCCGCGCCGCCCAGACTGCATCTGCGTGTTCGAATTCGCCAATGACAAAACGTTGGGAGG[T/G]GTGTGCGTCATCCTGGAGCTAAAGACATGCAAATCGATTTCTTCCGGGGACACGGCCAGCA | 48252 |
| 34 | TGGCCTCGCGGGCATCCCGGCCGATGCAGTCGCCCAGGTCGACGCGCGAGAGCGAGTACT[C/G]GGTCAGGTTGGTGGTGAAGGTGGTCGAGATGGCGTCGGAGGAGAAGCGGAAGGAGCCGCCG | 54967 |
| 35 | GAAAATTCTCGACATCATGCAGGAAGCACAGCTCCATGCGGACGTCCCCGCCGTACGTCT[C/G]CAGCCGGATCTGCTGGTGGTACGGACAGGGTCGGGCCAGACCCATGGTCTCGGTGAAAAAG | 58118 |
| 36 | CTGGTGGTACGGACAGGGTCGGGCCAGACCCATGGTCTCGGTGAAAAAGGCAGAGACGTC[A/T]CCCGTGGTCGCGAACGTTTCCAGGTGGCCCAGGAGCCGCTCCCCCTCGCGCCACGCGTACT | 58191 |
| 37* | GGCGGCCTCGGACGCGTTGGCGCTCGCGCCCGCGAACAACACGCGGCTCTTGACGCGCAG[T/C]TCCTTGGGAAACCCAAGGGTCACGCGGGCAACGTCGCCCTCGAAGCTGCTCTCGGCGGGGG | 60173 |
| 38 | TCCACCTCGCGATCCACCAGCTGCTTGATGTTGTTCACCACCGTGTGCAGGGCCTCGCGG[T/G]TGCCGATAATCGTCTCCAGCCTCCCCAGGGCCGTGGGCACCGCCTGGTCCACGTACTGCAG | 60657 |
| 39 | GCTGTGGTCCTTGGGGTCCGCCGGCCCGGCGTCGTCCACCTCGGTCAGGTGGAGGGCCGA[A/G]TTGGTGCTGAACACCATGGCGCCCACGAGGCCCGCGGCGCGCGCCAGGTACGCCCCGACGG | 61343 |
| 40 | CGCACAGCGCGTCCCCCGTCGTCTCGTGCTTTAGGTCGCAGGGCCGGGGCGCGTAGTCCG[A/C]GAAGCCAAAATGGCGGCGCGCCCGCTCGCAGAGCCGCGTCAGGTTGGGGGCCTGGGTGCTG | 62107 |
| 41 | CCGCGTCAGGTTGGGGGCCTGGGTGCTGGGGGCCAGGTGGCGGCCGCCGTGAAAGACGTA[A/G]ACGGACGGGCTGTAGTGCGAGGGCATAAGCTTGAGGGACACCGCGGTCCCCCCAAGGCCCG | 62201 |
| 42 | GCCGCCGGCAGAACTTCTACAACCCCCACCTCGCTCAGACCGGAACGCAGCCAAAGGCCC[T/C]CGGGCCGGCTCAGCGCCATACGTACTACAGCGAGTGCGACGAATTTCGATTTATCGCCCCG | 63478 |
| 43 | GGCCCGGAGGGCTTCTGGCCGCGTCGCTTGCGCCTGTGGGGCGGTGCGGACCATGCCCCC[A/G]AGGGGTTCGACCCCACCGTCACCGTCTTCCACGTGTACGACATCCTGGAGCACGTGGAACA | 63714 |
| 44 | GCTGGCCTTTCCGGTCGCGGAACGCCCGGAAGACCTCGTCATCCAGATCTCCTGTCTGCT[C/G]TACGACCTGTCCACCACCGCCCTCGAGCACATCCTCCTGTTTTCGCTCGGATCCTGCGACC | 64496 |
| 45 | CAGGCAGTTGTCGGCCACGGTCTGGTCCAGGCTGAAGGGGAGCGACACGGGGGTCGTCTT[T/C]ACCAGGGGCACGGAGAGCGAGCGCACGATGGCGATCTCCTCGGAGGGCGTCTGGGCGAGGG | 67627 |
| 46 | CACGGTCTGGTCCAGGCTGAAGGGGAGCGACACGGGGGTCGTCTTCACCAGGGGCACGGA[T/G]AGCGAGCGCACGATGGCGATCTCCTCGGAGGGCGTCTGGGCGAGGGCGGCGAAGAAGCCGC | 67642 |
| 47 | GGCGCGGGGGTGGCCAAACAGGACCCAGGGGTCGACTTCCATCTCCGTGATGGCGCACAT[C/G]GGATCGCAGAACATGTGCTTGAAGATGGCCTCGGGGCCCGCGGCCCGAAGCAGGCTCACGA | 68168 |
| 48 | TGTGCCCTGCAACCCCTACCTGCGCGTGCAGAACACCGGCGTTTCGGTGCTGTTTCAGGG[T/G]TTTTTTAACCGGCCCCACGGCGCCCCGGGGGGCGCGATCACGGCGGAGCAGACCAACGTGA | 70447 |
| 49 | CGGCGGCCCGGAGCCCGCGGCCGCGCGCGTCCACTCGCCCCCCTCCACCAGACATCCCTC[A/G]ATGGCCTCCGCGGACAGCACGTCGCGGGGCCCCACGTCGAAAAGAAGACTGAGAAACGACA | 80775 |
| 50 | TGTGGGGGGGGGCGGCGCGACGGCGGCCCGGACCAAGTGTATCGCGGCCGTTCCGTGGGG[T/C]GGCCCAACAGGCCCTTTAAACATTTGCGTATGCACCGGCCCAGCCAGTCGGACACCGGAAC | 84897 |
| 51 | GGGGGGCCCTGGGGCGGCGTTCCTGTACTTGGTCTTCACCTACCGACAGTGCCGGGACCA[A/G]GAGCTCTGTTGCGTGTACGTGGTCAAGAGCCAGCTCCCCCCGCGCGGACTGGAGGCGGCCC | 86062 |
| 52 | ACAACAGGTGGGTGCTTCGGGGACTTGACGGTCGCCACTCTCCTGCGAGCCCTCACGTCT[T/C]CGCCCACCGATTCCTGTTGCGTTCCTGTCGGCCGGTGCTGTCCTGTCGACAGATTGTTGGC | 86880 |
| 53 | GCCTGATCCGCCACCTCGACGGCGAGAAAAACGTCACCTGGTCCCTGTTCGACCGGGACA[C/G]CAGCATGTCGCTCGCCGACTTTCACGGCGAGGAGTTCGAGAAGCTGTACGAGCACCTCGAG | 89230 |
| 54* | AGCGATCCAGTGGGGTCTGCAACCTGGGAAGCGTGAATCTGGCCCGATGCGTCTCCAGGC[A/G]GACGTTTGACTTTGGGCGGCTCCGCGACGCCGTGCAGGCGTGCGTGCTGATGGTGAACATC | 89539 |
| 55* | CTTTAAGCGCAGCATGTACCGGGCCGGCCGCTTTCACTGGGAGCGCTTTTCGAACGCCAG[C/G]CCGCGGTACGAGGGCGAGTGGGAGATGCTACGCCAGAGCATGATGAAACACGGCCTGCGCA | 89891 |
| 56 | CCACTCGCGCGTCTACAACATCATCCAGCTGGTGCTCTTTCACAACAACGACCAGGCGCG[C/G]CGCGCCTATGTGGCCCGCACCATCAACCACCCGGCCATTCGCGTCAAGGTGGACTGGCTGG | 90969 |
| 57 | GCCCATCAGCAGGAGATCGGTATCCGTGGTATGCACGTACGCGACCGTGTTGGTATGATA[C/G]AGGTTCGCGCAGGCGTCGTCGGCCTCCAGCTGACCCGAGTTGATGTAGGCGTACCCCAGCG | 92706 |
| 58 | CGGTGTCGGCGGTGTTTCTTTTAAAACCCCAGCGGGTCTGCCTGAACTGGCTCGGCCGGA[C/G]CCCGGGTTCCTCGACCGGGAGCTTGGCGTCCCAGGACTCTCGGGCCGGCCCGACCGACAGC | 94787 |
| 59 | CCTCGACCGGGAGCTTGGCGTCCCAGGACTCTCGGGCCGGCCCGACCGACAGCCAGGACT[T/C]CTCCTCCGAGCCGGACGCGGGCGACCGCGGCGCCCCAGAAGAAGAAGGCCTCGAGGGCCAG | 94856 |
| 60 | CCCGGTACGGCTCGCGGGTGCAAATCCGATGCCGGTTTCCCAACTCCACCCGCACGGAGT[T/C]CCGCCTCCAGATCTGGCGTTATGCCACGGCGACGGACGCCGAGATCGGAACGGCGCCTAGC | 97382 |
| 61 | CTGCGCTAGGATGGCGCGGCTCAGCTGGCCGCCCGACAGCTCCACCTCGCCGAGCGCCTG[A/C]TTGGCGGCCGACGCGTAGTGCCGGATGTAGTCGTAGTGCGGGTCGCTGGCGAGCCCGTCTA | 101305 |
| 62 | CGCGGGAGCGTCATCGTCGTCCGGGAGGTCGAGCAGGCCCTCGATTGTCGATCCGTAATT[A/G]TTTCTGGTCCGCCCGCGGCTATACGCGTGCTCCCGCATGACGGACTCGCCCTCCGAGGTCG | 104696 |
| 63 | GCGGAGGGGCGGCTGGGGTGTTCTTGGGACCCCCGGCTGGCCTGGGGGGCGGTGGCGAAA[T/C]CCCGTCCGCGTCCGCAAACAGATCGTCGACCAACAGGTCCATGGGGGCGGTTGGGTCCGGG | 105760 |
| 64* | CGCCGAACGACGCGGTCGCGCGGTATCCCGGGACTCGTCGTCGTCCGAAGACGAGTCCCG[A/G]TAGAGGGCATACCCAGCCTCGTCATAATGGAGAAAGCGAACCTCGCCCCTCGGGCGCGCGC | 106985 |
| 65 | CATGGTCTATCGGAGACACCGGGGACGCCCGTGCGGATCACAGGGAAGGCGTCGGCGAAG[C/G]AGGCAGAGAGCGTCGGAAGGCGGCGAGGGAGGGAAAGAGGGAGACCGGCGGGGTACGGGAG | 107243 |
| 66 | ACACGCCCACCCCTTCCCAACAGGGCGGGCTCAGGCTGACCCGGCGGCCAGTGCCCGCTG[A/G]CATATCTGATACACGTGCGCGATCATACATACGCCCATCGAGGTCATGCCTAGATAAAAGG | 107554 |
| 67 | CGGGGACTCCGAATGGGCCGTGGGCCGCGTCTCTCTGGGCCTGCGAATGGCAATGCCGCG[T/G]GACTTCTGCGCGATTATTCACGCCCCCGCGGTATCCGGCCCCGGGCCCCACGTGATGCTCG | 108013 |
| 68 | GCCCCGAACGGGACGCGCGGGTTTGCCCCCGGGGCCCTCCGGGTCGACGTGACGTTTCTG[A/G]ACATCCGGGCCACCCCCCCGACCCTCACCGAGCCGAGCTCCCTGCACCGGTTTCCGCAGTT | 108182 |
| 69 | CTCACAAACTGCCTGCTGGGGGCCGAGCCGTTGTATATATTCAGCTACGACGCGTACCGG[T/C]CCGATGCGCCCAATGGCCCCACGGGCGCGCCCACCGAACAGGAGAGGTTCGAGGGGAGCCG | 110078 |
| 70 | GACGACGTCGCCGTGCTCCAAGACGCCCTGGGCCGCGGGACCCCATTGCTCCCGGCCCAC[A/G]TCACAGCAACTCTGGACTTGGAGGCGACGTTTGCGCTCCACGCTAACATCATCATGGCTCT | 110339 |
| 71 | GCGGCCGCTGGGTTTCCGCTGTATGTGGAGCGCCGCATCGCCGCCGACGTACGCGAGACC[A/G]GCGCGCTGGAGAAGTTCATCGCCCACGATCGCAGCTGCCTGCGCGTGTCCGACCGGGAATT | 110909 |
| 72* | CATCGCCGCCGACGTACGCGAGACCGGCGCGCTGGAGAAGTTCATCGCCCACGATCGCAG[T/C]TGCCTGCGCGTGTCCGACCGGGAATTCATTACGTACATCTACCTGGCCCACTTTGAGTGCT | 110944 |
| 73 | CACGGAGCAGCCCTCGCCCCTGGGTCGGGAGGCGGTGGAACAGTTCTTCCGGCACGTGCG[T/C]GCCCAGCTGAACATCCGCGAGTACGTAAAGCAAAACGTCACCCCCAGGGAAACCGCCCTGG | 111133 |
| 74 | CATCGTCTTTGGTGCCAGTCCGCTCCACCGATGTATTTACGCGGTGCGCCCCGCCGGGGC[A/G]CACAACGATACCGCCCTCGTGTGGATGAAGATAAACCAGACGCTGTTGTTTCTGGGCCCGC | 113199 |
| 75 | CGCTGTTGTTTCTGGGCCCGCCGACCGCCCCCCCCGGCGGGGCATGGACCCCCCACGCCC[A/G]CGTCTGCTACGCCAATATCATCGAAGGTCGGGCCGTGTCCCTCCCGGCCATCCCCGGCGCC | 113300 |
| 76 | GGGGCGCCTGCGCGATCACACACCCCCTGTTTCTAACAATCACCACCTGGTGTTTCGTGT[C/G]CATCATCGCCCTGACGGAGCTGTATTTCATCCTGCGGCGGGGCTCGGCCCCCAAAAACGCG | 113840 |
| 77* | TTTCTAACAATCACCACCTGGTGTTTCGTGTCCATCATCGCCCTGACGGAGCTGTATTTC[A/G]TCCTGCGGCGGGGCTCGGCCCCCAAAAACGCGGAACCAGCGGCCCCCAGGGGGCGCTCCAA | 113869 |
| 78* | CCACCTGGTGTTTCGTGTCCATCATCGCCCTGACGGAGCTGTATTTCATCCTGCGGCGGG[A/G]CTCGGCCCCCAAAAACGCGGAACCAGCGGCCCCCAGGGGGCGCTCCAAAGGGTGGTCGGGC | 113882 |
| 79 | AAATACACGACCCGCCTCGGGCCTACGCACCCTCGCACGTCGCATGCAAATTAAAATCGT[A/G]CACAGAGCCGATCCGGCCTCGGGTCTGCTTGCCCCTCCCCCGGCCCAGCACAGGCAGGCTC | 114189 |
| 80 | TGCAAATTAAAATCGTGCACAGAGCCGATCCGGCCTCGGGTCTGCTTGCCCCTCCCCCGG[T/C]CCAGCACAGGCAGGCTCGTCCGACTTCCGCATACACCCCACCCTACCGCGTGCTTCCGCAC | 114233 |
| 81* | GGCAGGCTCGTCCGACTTCCGCATACACCCCACCCTACCGCGTGCTTCCGCACCCCCGCC[T/C]ACGCGTGTACGCGAAGGCGGACCCAGACCTGCCGTATGCTAATTAAATACATAAAACCCAC | 114302 |
| 82 | CTATCGCCCCCTTATATGTGCACGGCAAATACTTCTACTGCAACTCCCTATTTTAGGCAA[A/G]AATAAACATATTGACGTCAACCCAAGTGGTTCCGTGTGATGTTCTTGGCGCGCGCGGCGGG | 116167 |
| 83 | CCCATTCAGCTGCGGAACCATCAAGGACGTCTCCGGTGCATCCCCCGCGGGGGAATACAC[T/G]ATAAACGGTATCGTGTACCACTGTCACTGTCGGTATCCGTTCTCCAAAACCTGCTGGCTCG | 116769 |
| 84 | CAGACGCGGTGTGAGTTTGTGGGTTATAGGAACCCGGTAAATACCACGCGACGAACCAGC[A/G]TGTGTGTTAACGCAACTTTTATTCGTTGTATCGCGGGAGGGGGGAAGCTTACCGCCAAAGG | 117061 |
| 85 | GTCGGAGCTCGACGCACAGCGGGCCGCGCGTTGGGCCCGGTACAGCTCTCGCGAATTGAC[A/G]AGCGGGGGTCGCCACGTGCGCGAGCTTTGCACGCGGGGTTGGTCGGCCGGCCCCACGGACC | 117272 |
| 86 | CCCCCGCAAATCCAAGCGACCCCGAATCAACCTGCGATTAACGAGCTCCCCCGACCGGCG[T/C]GCGGGTGTGGTTTTCCCCGAGGTGTGGAGAAGCGACAGACCTATCCGCGCGGCGCAACCCC | 134145 |
| 87 | GATGCGGAGCGGAGCCGCCTGGACGCTTGATCTGCATTACATACGCCAGTGCGTCAACCA[A/G]CTCTTTCGGATCCTGCGTGCCGCCCCGAACCCGCCCGGCAGCGCCAACCGCCTGCGCCACC | 134331 |
| 88 | TCCGGCCATGAGCGCGGGACCCCCAGCCCGGTGTGTTTGCCAAACGAAAAATAAACGCCC[T/G]ACAAGAAAGCTTTTGTGTCTGAGTGTCTGGTTTTTCTGGGGGTGGAGGAAGGAACGACAAA | 135035 |
| 89 | CGCGTCGTTGGTCTTCGTGGCGATGAAGCGAAACCCCAGCCGGGTTTTTTGTGCGTACTC[T/G]AAAAACGGCACACACAGGTCCGCCGCCCCGACCACCCACAGGTGGTATAGCCGGTGGGGGC | 135633 |
| 90 | GTCGACGAGTTTCCGACGCACGCGGAATCGCGCCTCACCGCGCACTACCGCTCGCGGGCG[A/G]CCGGGAACAATCGTCCGGCGTGGACCCGACCGGCGTGGACCCGCTACTACAAGATCCACAC | 137613 |
| 91 | CACCGCGCACTACCGCTCGCGGGCGGCCGGGAACAATCGTCCGGCGTGGACCCGACCGGC[A/G]TGGACCCGCTACTACAAGATCCACACAGACGTCGAATATCTCATATGCAAAGCCCTTACCT | 137648 |
| 92 | TGGTTTTGCTGGCGCCGCCGGTACGCGGATTTGGCGCACCCAACGCAACGTATGCGGCCC[A/G]TGTGACGTACTACCGGCTCACCCGCGCCTGCCGTCAGCCCATCCTCCTTCGGCAGTATGGA | 138210 |
| 93 | CGATTCGCCTACGTCCGCTCCGGAGAAGACGCCCCTCCCTGTGTCGGCCACCGCCATGGC[A/G]CCCTCAGTCGACCCAAGCGCGGAACCGACCGCCCCCGCAACCACTACTCCCCCCGACGAGA | 138997 |
| 94 | GCGGGCCAATTTGCCCCATGATTTTTCGCCTTTCTGGCCTTGCCCCCACCCCATCGCCCC[C/G]ATTGTGTGTCGGGTGCCCGGGGTACAGCAGCTATGGAGCGGTCGGTAATATAACTTTGGTT | 140609 |
| 95 | CCGCCGCCCCCAGCAACCCGGGCCTGATCATCGGCGCGCTGGCCGGCAGTACCCTGGCGG[T/C]GCTGGTCATCGGCGGTATTGCGTTTTGGGTACGCCGCCGCGCTCAGATGGCCCCCAAGCGC | 142073 |
| 96 | CCCCCTCGCACCAGCCATTGTTTTACTAGAGGAGTTTCCCCGCTCCCGTGTACCTCTGGG[T/C]CCGTGTGGGAGGGTGGCTGGGGTATTTGGGTGGGACTTGGACTCCGCATAAAGGGAGTCTC | 142229 |
|  |  |  |
|  |  |  |
| *SNPs that were removed from analysis due to poor performance (call rate<90%). | |  |
