# Supplementary material for: Dual-strain genital herpes simplex virus type 2 (HSV-2) infection in the US, Peru, and 8 countries in sub-Saharan Africa: A nested cross-sectional viral genotyping study
Source: PLoS Med. 2017 Dec 27;14(12):e1002475. doi: 10.1371/journal.pmed.1002475 (PMC5744910; doi:10.1371/journal.pmed.1002475)
Supplement: S2 Table — (DOCX) [file pmed.1002475.s009.docx]

| Pair name | Specimen number | Country | Sex | HIV status | Mismatches by Array | Mismatches by sequence | Genbank accession |
| --- | --- | --- | --- | --- | --- | --- | --- |
| 2_a | 2003-18061 | USA | M | Positive | 26 of 84 | 268 of 121106 | MF510298 |
| 2_b | 2007-22031 | USA | M | Positive | 26 of 84 | 268 of 121106 | MF510341 |
| 3_a | 2005-10915 | USA | F | Negative | 21 of 85 | 28 of 122363 | MF510362 |
| 3_b | 2005-37403 | USA | F | Negative | 21 of 83 | 28 of 122363 | MF621254 |
| 4_a | 2006-46602 | Peru | F | Positive | 19 of 85 | 235 of 122404 | MF621255 |
| 4_b | 2006-46638 | Peru | F | Positive | 19 of 83 | 235 of 122404 | MF510320 |
| 5_a | 2006-21876 | Zimbabwe | F | Negative | 29 of 85 | 330 of 122380 | MF510348 |
| 5_b | 2007-38113 | Zimbabwe | F | Negative | 29 of 85 | 330 of 122380 | MF621256 |
| 6_a | 2006-20641 | Zambia | F | Negative | 24 of 85 | 274 of 122529 | MF510309 |
| 6_b | 2006-23142 | Zambia | F | Negative | 24 of 85 | 274 of 122529 | MF510302 |
| 7_a | 2006-46019 | Zambia | F | Negative | 11 of 85 | 129 of 122406 | MF510322 |
| 7_b | 2006-46118 | Zambia | F | Negative | 11 of 85 | 129 of 122406 | MF510269 |
| 9_a | 2009-2139 | Zambia | F | Positive | 11 of 82 | 130 of 122529 | MF510343 |
| 9_b | 2009-2258 | Zambia | F | Positive | 11 of 78 | 130 of 122529 | MF510287 |
| 10_a | 2009-393 | Zambia | M | Positive | 24 of 85 | 280 of 122386 | MF510325 |
| 10_b | 2009-2254 | Zambia | M | Positive | 24 of 85 | 280 of 122386 | MF510330 |
| 11_a | 2009-2246 | Zambia | F | Positive | 19 of 82 | 268 of 122485 | MF510312 |
| 11_b | 2009-2265 | Zambia | F | Positive | 19 of 85 | 268 of 122485 | MF510361 |
| 12_a | 2009-3495 | Uganda | M | Positive | 14 of 85 | 413 of 122357 | MF510280 |
| 12_b | 2009-3539 | Uganda | M | Positive | 14 of 84 | 413 of 122357 | MF510296 |
| 13_a | 2009-2222 | Botswana | F | Positive | 19 of 85 | 174 of 122436 | MF510299 |
| 13_b | 2009-3425 | Botswana | F | Positive | 19 of 84 | 174 of 122436 | MF510289 |
| 14_a | 2009-4463 | Kenya | F | Positive | 18 of 85 | 326 of 122263 | MF621257 |
| 14_b | 2009-4532 | Kenya | F | Positive | 18 of 84 | 326 of 122263 | MF510275 |
| 15_a | 2009-406 | Tanzania | F | Positive | 15 of 85 | 287 of 122501 | MF510303 |
| 15_b | 2009-3488 | Tanzania | F | Positive | 15 of 84 | 287 of 122501 | MF510347 |
| 17_a | 2012-18420 | Uganda | M | Positive | 11 of 85 | 366 of 122309 | MF510337 |
| 17_b | 2012-20191 | Uganda | M | Positive | 11 of 83 | 366 of 122309 | MF510358 |

S2 Table. Baseline characteristics and GenBank number for samples that underwent high throughput sequencing.

*4 pairs which were identified as superinfected but could not be sequenced are not included in this table.
