# Supplementary material for: Dual-strain genital herpes simplex virus type 2 (HSV-2) infection in the US, Peru, and 8 countries in sub-Saharan Africa: A nested cross-sectional viral genotyping study
Source: PLoS Med. 2017 Dec 27;14(12):e1002475. doi: 10.1371/journal.pmed.1002475 (PMC5744910; doi:10.1371/journal.pmed.1002475)
Supplement: S3 Table — (DOCX) [file pmed.1002475.s010.docx]

|  | Univariable analysis | | Multivariabe analysis | |
| --- | --- | --- | --- | --- |
| Characteristic | Risk ratio (95% CI) | p-value | Risk ratio (95% CI) | p-value |
| Male | 0.65 (0.29, 1.45) | 0.30 | - | - |
| Age in decades | 0.95 (0.67, 1.34) | 0.75 | - | - |
| Continent  North America (US)  South America (Peru)  Africa | Ref  2.34 (0.60, 9.05)  5.58 (2.23, 13.93) | Ref  0.22  0.0003 | Ref  1.87 (0.47, 7.52)  4.38 (1.69, 11.36) | Ref  0.38  0.0025 |
| Lifetime number of sexual partners,  each additional ten | 0.99 (0.84, 1.17) | 0.90 | - | - |
| HIV seropositive | 3.77 (1.73, 8.22) | 0.0001 | 2.81 (1.25, 6.34) | 0.013 |
| Homosexual or bisexual | 0.54 (0.20, 1.43) | 0.22 | - | - |
| At least 3 years between samples | 1.61 (0.39, 6.57) | 0.51 | - | - |

S3 Table. Univariable and multivariable sensitivity analysis including 8 pairs that were excluded from the analysis due to inability to confirm that they were collected from the same person.
