# Supplementary material for: Dual-strain genital herpes simplex virus type 2 (HSV-2) infection in the US, Peru, and 8 countries in sub-Saharan Africa: A nested cross-sectional viral genotyping study
Source: PLoS Med. 2017 Dec 27;14(12):e1002475. doi: 10.1371/journal.pmed.1002475 (PMC5744910; doi:10.1371/journal.pmed.1002475)
Supplement: S1 Text — (DOC) [file pmed.1002475.s011.doc]

| **Table 4.** Probability calculations to determine number of SNPs to genotype | | | | |
| --- | --- | --- | --- | --- |
|  | Probability of observing a difference between strains if the minimum prevalence of the SNP = X | | | |
| # of SNPS | X=10% | X=20% | X=25% | X=30% |
| 10 | 26% | 62% | 76% | 85% |
| 15 | 45% | 83% | 92% | 96% |
| 20 | 61% | 93% | 98% | 99% |
| 50 | 97% | >99% | >99% | >99% |

**C.4. ANALYSIS AND POWER CALCULATIONS.**

*Determination of multiple-strain infection. The 48 samples from Phase I will provide a preliminary estimate of the prevalence of multiple strain infection, and will inform power calculations to determine how many samples we need to genotype to achieve our epidemiologic goals; based on published studies, we estimate that we will need to genotype 600 persons. As shown in* ***Table 4****, the probability of detecting different strains increases with the number of SNPs that are genotyped as well as the prevalence of each individual SNP in the population. Based on this, we will sequence up to 5 loci containing 15-20 SNPs, each with a population prevalence of ≥30%, which will give ≥96% probability of detecting multiple strain infection. We use Bayes’ reasoning to calculate the degree of certainty with which we can declare single or multiple strain infection, using standard transformations of the conditional probabilities of variant SNP detection when infection status is known. These computations are identical to computing PPV/NPV from sensitivity and specificity.* ***Table 5*** *demonstrates that if we use 2 SNPs to declare mismatch, we will have ≥98% probability of correctly identifying mismatches, if the prevalence of multiple strain infection is ≥10%.* We estimate that multiple strain infection has a prevalence of ~10%, based upon data from previous studies (**Table 1**). We will have ≥99% probability of identifying identical strains given a match at all SNPs. If strains differ at one SNP only, we

| **Table 5.** Rationale for use of 2 SNPs to declare a mismatched strain | | | |
| --- | --- | --- | --- |
| Prevalence of multiple  strain infection | Probability of nonidentical strains given a mismatch, if X differing SNPs are required to declare mismatch | | |
|  | X=1 | X=2 | X=3 |
| 10% | 66% | 98% | >99% |
| 20% | 81% | 99% | >99% |
| 30% | 88% | 99% | >99% |
| 40% | 92% | >99% | >99% |

will consider examining other loci to determine whether two strains are present, such that we will be able to make a call with ≥95% probability. Drs. Wald and Koelle will independently assess whether multiple strain infection is present in each pair of specimens from an individual. In case of disagreement, we will request input from another HSV virologist (Dr. Keith Jerome). Of primary interest is a precise estimate for prevalence of multiple strain infection (**Table 6**), which we will be able to provide definitively with our cohort. The degree of precision will depend on both the number of participants we study and the prevalence of multiple strain infection. We will use multivariate models to calculate relative risks of the identified potential risk factors for multiple strain infection. With a sample size of 600 persons, we will be able to efficiently address each of our hypotheses based upon the power calculations shown in **Table 7**. For instance, if baseline prevalence

| **Table 6.** Point estimates and confidence intervals for various estimates of prevalence of multiple strain infection | | | |
| --- | --- | --- | --- |
| Number of participants | Confidence interval for prevalence X | | |
|  | X=5% | X=10% | X=15% |
| 600 | (3.5%, 7.1%) | (7.8%, 12.7%) | (12.4%, 18.1%) |
| 400 | (3.2%, 7.6%) | (7.4%, 13.3%) | (11.8%, 18.8%) |
| 150 | (2.5%, 9.9%) | (6.1%, 15.9%) | (10.1%, 21.6%) |

of multiple strain infection is 5%, with 300 each men and women, we will have 88% power to detect a 2.5-fold increase and 98% power to detect a 3-fold increase in multiple strain infection in women. We will also have 88% power to detect a 2.5-fold increase in multiple strain infection in persons with ≥10 lifetime partners as compared to fewer partners. If we assume higher baseline prevalence of multiple strain infection (e.g. 15%), our power increases, such that with a sample size of 400, we have 86% power to detect a 2-fold increase in multiple strain infection in people from Africa and Peru as compared to the USA.

| **Table 7. Power to detect increase in prevalence of multiple strains from XX participants. Scenarios with less than 80% power have been shaded grey.** | | | | | | | | |
| --- | --- | --- | --- | --- | --- | --- | --- | --- |
| Variable | % with  variable  of interest | Total  sample  size | Power to detect X-fold increase if prevalence=**5%** | | | Power to detect X-fold increase if prevalence=**15%** | | |
|  |  |  | X=2 | X=2.5 | X=3 | X=2 | X=2.5 | X=3 |
| Women, number of sex partners | 50% | 600 | 58% | **88%** | **98%** | **99%** | **99%** | **99%** |
|  |  | 400 | 40% | 70% | **89%** | **94%** | **99%** | **99%** |
|  |  | 150 | 12% | 25% | 41% | 51% | **84%** | **98%** |
| HIV status, geography | 25% | 600 | 51% | 79% | **93%** | **96%** | **99%** | **99%** |
|  |  | 400 | 36% | 62% | **81%** | **86%** | **99%** | **99%** |
|  |  | 150 | 12% | 25% | 39% | 44% | 75% | **93%** |
